# Race/ethnicity in systemic AL amyloidosis: perspectives on disease and outcome disparities

**DOI:** 10.1038/s41408-020-00385-0

**Published:** 2020-11-10

**Authors:** Andrew Staron, Lawreen H. Connors, Luke Zheng, Gheorghe Doros, Vaishali Sanchorawala

**Affiliations:** 1 Amyloidosis Center, Boston University School of Medicine and Boston Medical Center, 1 Boston Medical Center Place, Boston, MA USA; 2 Section of Hematology and Oncology, Boston University School of Medicine and Boston Medical Center, 1 Boston Medical Center Place, Boston, MA USA; 3 Department of Pathology and Laboratory Medicine, Boston University School of Medicine and Boston Medical Center, 1 Boston Medical Center Place, Boston, MA USA; 4 Department of Biostatistics, Boston University School of Public Health, 715 Albany Street, Boston, MA USA

**Keywords:** Risk factors, Cancer epidemiology

## Abstract

In marked contrast to multiple myeloma, racial/ethnic minorities are underrepresented in publications of systemic light-chain (AL) amyloidosis. The impact of race/ethnicity is therefore lacking in the narrative of this disease. To address this gap, we compared disease characteristics, treatments, and outcomes across racial/ethnic groups in a referred cohort of patients with AL amyloidosis from 1990 to 2020. Among 2416 patients, 14% were minorities. Non-Hispanic Blacks (NHBs) comprised 8% and had higher-risk sociodemographic factors. Hispanics comprised 4% and presented with disproportionately more BU stage IIIb cardiac involvement (27% vs. 4–17%). At onset, minority groups were younger in age by 4–6 years. There was indication of more aggressive disease phenotype among NHBs with higher prevalence of difference between involved and uninvolved free light chains >180 mg/L (39% vs. 22–33%, *P* = 0.044). Receipt of stem cell transplantation was 30% lower in Hispanics compared to non-Hispanic White (NHWs) on account of sociodemographic and physiologic factors. Although the age/sex-adjusted hazard for death among NHBs was 24% higher relative to NHWs (*P* = 0.020), race/ethnicity itself did not impact survival after controlling for disease severity and treatment variables. These findings highlight the complexities of racial/ethnic disparities in AL amyloidosis. Directed efforts by providers and advocacy groups are needed to expand access to testing and effective treatments within underprivileged communities.

## Introduction

The incidences of multiple myeloma and its precursor state, monoclonal gammopathy of undetermined significance (MGUS), differ drastically by race/ethnicity. Compared to Whites, rates are over twofold higher in Blacks, slightly higher in Hispanics, and markedly lower in Asians and Pacific Islanders^1–5^. Systemic light-chain (AL) amyloidosis belongs to the spectrum of plasma cell disorders, which include multiple myeloma and MGUS^6^. Accordingly, it would be expected to affect various racial/ethnic groups in similar disproportions. The largest population-based studies of AL amyloidosis, however, are composed almost entirely of White patients^7,8^. The basis for this observation is uncertain. Inequitable access to diagnostic testing and fewer referrals to tertiary centers among disadvantaged groups may be important factors—rather than protective biology.

The Amyloidosis Center at Boston University is situated within a large safety net hospital for the underserved, offering a unique opportunity to evaluate racial/ethnic disparities. In a prior report from our center, emerging recognition of amyloid diseases among underrepresented groups was noted: Blacks doubled in proportion over a three-decade period from 5 to 10% of patients with systemic AL amyloidosis^9^. To date, little is known about the unique hematologic and organ disease features of this disease in racial/ethnic minorities, and how clinical outcomes may differ. Based on US death certificate data from a national database, mortality due to cardiac amyloid diseases—including both AL and transthyretin-related (ATTR) amyloidosis—was found to be highest among Black men^10^. In multiple myeloma, racial/ethnic identity has an impact on clinical outcomes, largely due to disproportionate use of effective treatments (i.e., stem cell transplantation)^11–16^. With equal access to treatments, survival among Black patients with multiple myeloma is similar or potentially superior compared to Whites^2,17–20^.

Understanding the health obstacles that marginalized patients with hematologic disorders face is necessary to ensure care of equal quality. This article brings new insights on the role of race/ethnicity in systemic AL amyloidosis, including inequities in disease presentation and receipt of aggressive treatment with stem cell transplantation.

## Methods

### Data collection and patients

Data were abstracted from a database of consented patients seen at the Amyloidosis Center at Boston University School of Medicine and Boston Medical Center, which is maintained prospectively under the approval of the Institutional Review Board in accordance with the Declaration of Helsinki and the Health Insurance Portability and Accountability Act Guidelines (ClinicalTrials.gov Identifier: NCT00898235). The diagnosis of AL amyloidosis was predicated on positive staining of tissue with Congo red and evidence of plasma cell clonality in the setting of appropriate systemic syndrome. Multiple myeloma-associated AL amyloidosis signified cases that also fulfilled the International Myeloma Working Group criteria for multiple myeloma, including both myeloma-defining end-organ features and clonal bone marrow plasma cell requirements, which changed over the course of the study period. In view of the high prevalence of hereditary ATTR amyloidosis among individuals of African ancestry^21,22^, self-reported Black patients underwent screening for the transthyretin V122I genetic variant using isoelectric focusing^23^ and, when appropriate, mutation confirmation by direct DNA-sequencing^21^. Mass spectrometric analysis of tissue confirmed the presence of AL rather than ATTR amyloidosis in cases where the V122I allele was detected.

Sociodemographic (educational level, marital status) and lifestyle (alcohol consumption, smoking status, body mass index (BMI)) factors, along with baseline hematologic and organ characteristics, were stratified according to self-identified race/ethnicity. Groups were mutually exclusive and included non-Hispanic White (NHW, i.e., White alone), non-Hispanic Black (NHB, i.e., African American, Afro-Caribbean, or Black alone), Hispanic (i.e., Hispanic or Latinx, any race), and non-Hispanic other (NHO, i.e., Asian, Pacific Islander, American Indian, Alaskan Native, more than one race, or unidentified).

### Staging and response assessment

Organ involvement was defined according to the consensus criteria of the International Society of Amyloidosis^24^. Cardiac staging was established on brain natriuretic peptide (BNP, threshold > 81 pg/mL) and troponin-I (threshold > 0.1 ng/mL) with stages I, II, and III defined according to none, one or both markers above threshold^25^. Stage IIIb was classified if BNP was also >700 pg/mL. Similarly, renal staging was based on estimated glomerular filtration rate by Chronic Kidney Disease Epidemiology Collaboration (CKD-EPI) creatinine equation (threshold < 50 mL/min per 1.73 m^2^) and 24-h proteinuria (threshold > 5 g/24 h)^26^. For patients who returned to our center for follow-up evaluation, interim treatments received were recorded, and hematologic response defined according to a validated criterion using immunofixation electrophoresis and serum free light-chain ratio^27^.

### Statistical analysis

Where present, incomplete data were reviewed and determined missing at random (i.e., due to variable entry not yet built into the database, unavailability of laboratory test, or data point not ascertained during history taking from patient) and a complete-case approach was taken. The number of complete cases (*N*) for those variables with missing data was indicated in the tables. For baseline descriptors, categorical variables were presented as proportions and differences assessed by the *χ*^2^ test of independence. Continuous data were presented as medians with interquartile ranges (IQRs) and comparisons made by the one-way analysis of variance test. Multivariable logistic regression was used to evaluate the confounding effect of sociodemographic and physiologic variables on stem cell transplantation utilization. Overall survival was defined as time from tissue diagnosis to date of death from any cause (event) or last follow-up (censored). Unadjusted survival curves were plotted by Kaplan–Meier method. Hazard ratios (HRs) for death and 95% confidence intervals (CIs) were estimated using multivariate Cox proportional hazards regression models in order to determine the effect of race/ethnicity on mortality while adjusting for relevant confounders. Initially, only demographic variables were included (model 1). Sociodemographic and lifestyle factors were then added (model 2), followed by physiologic factors (model 3). Lastly, stem cell transplantation treatment was included (model 4). *P* values were two-sided with <0.05 considered statistically significant. Computations were performed with the SAS version 9.4 software.

## Results

**Fig. 1:**
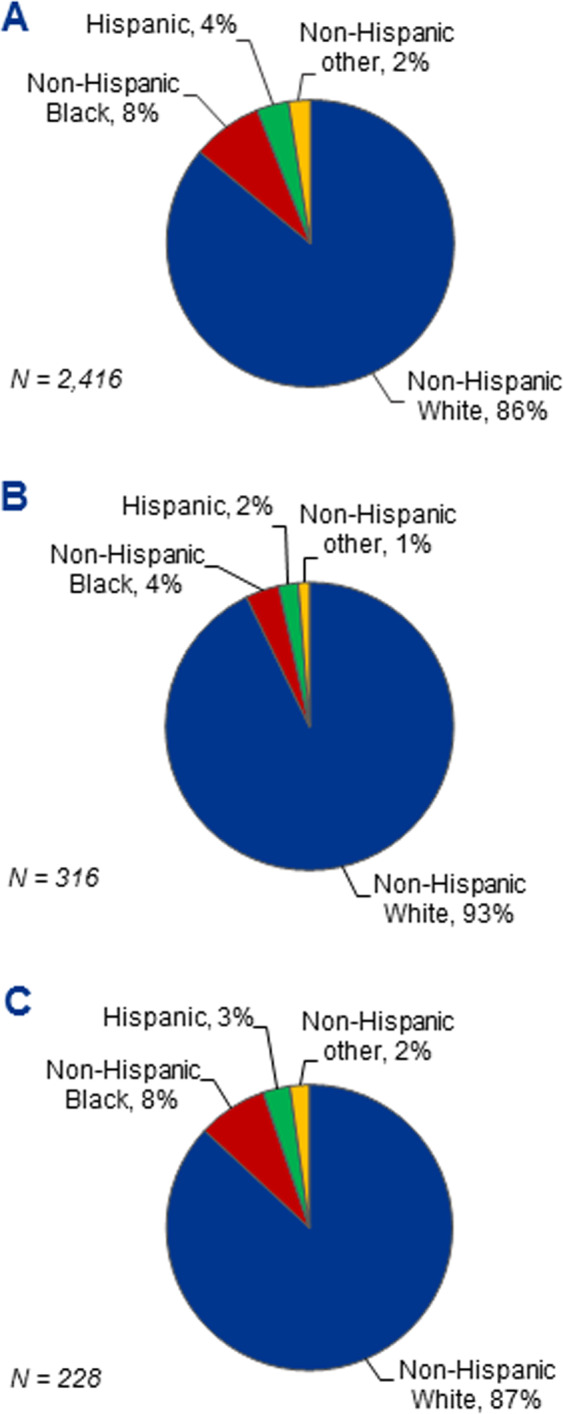
Racial/ethnic composition of AL amyloidosis. **a** The total cohort is shown in comparison to the study cohorts of **b** 12 SCT and **c** 18 non-SCT clinical trials conducted at the Amyloidosis Center.

A total of 2416 patients with systemic AL amyloidosis were evaluated between January 1, 1990 and January 1, 2020, including 2078 (86%) NHW, 192 (8%) NHB, 87 (4%) Hispanic, and 59 (2%) NHO individuals (Fig. 1a). Underrepresented minorities (NHB, Hispanic, and NHO) comprised an increasing proportion of the population over time: 8% in 1990–1999; 13% in 2000–2009; and 19% in 2010–2020. NHB and Hispanic patients were more likely to be local referrals (i.e., from the state of Massachusetts, Table 1). Median age was significantly different between groups (*P* < 0.001) with NHOs diagnosed the youngest at 56 years and NHWs the oldest at 62 years. Sex distribution was similar across groups with males comprising 54–59% of the cohort.

**Table 1:** Sociodemographic and lifestyle factors by race/ethnicity.

	NHW	NHB	Hispanic	NHO	*P* value
*N* (total = 2416)	2078	192	87	59	
Median age, years (IQR)	62 (54–69)	58 (50–66)	58 (52–67)	56 (45–65)	<0.001
Sex, *n* (%)					
Male	1229 (59%)	114 (59%)	48 (55%)	32 (54%)	
Female	849 (41%)	78 (41%)	39 (45%)	27 (46%)	0.778
Geographic origin, *n* (%)					
Massachusetts (local)	371 (18%)	47 (25%)	28 (32%)	8 (14%)	<0.001
Outside of New England	1358 (65%)	131 (68%)	52 (60%)	47 (80%)	0.068
Educational level (*N* = 1955), *n* (%)					
Postsecondary	1261 (75%)	92 (61%)	36 (56%)	41 (76%)	
High school or less	425 (25%)	58 (39%)	29 (45%)	13 (24%)	<0.001
Marital status (*N* = 2249), *n* (%)					
Married	1526 (80%)	128 (67%)	65 (75%)	46 (81%)	
Single	124 (6%)	35 (18%)	9 (10%)	3 (5%)	
Separated or widowed	265 (14%)	27 (14%)	13 (15%)	8 (14%)	<0.001
Alcohol consumption (*N* = 2346), *n* (%)					
No use	855 (42%)	123 (67%)	48 (55%)	40 (68%)	
Occasional use	927 (46%)	52 (28%)	34 (39%)	17 (29%)	
Moderate-to-heavy use	234 (12%)	9 (5%)	5 (6%)	2 (3%)	<0.001
Smoking status (*N* = 2267), *n* (%)					
Never smoker	1097 (57%)	113 (61%)	50 (60%)	43 (73%)	
Current or former smoker	843 (43%)	72 (39%)	33 (40%)	16 (27%)	0.054
Median pack years (IQR)	15 (4–35)	10 (2–30)	20 (4–30)	8 (1–30)	0.117
BMI class (*N* = 2044), *n* (%)					
Normal (18.5–24.9)	656 (38%)	68 (39%)	28 (37%)	34 (64%)	
Overweight (25–29.9)	693 (40%)	50 (29%)	35 (46%)	17 (32%)	
Obese (≥30)	391 (22%)	57 (33%)	13 (17%)	2 (4%)	<0.001
Median BMI, kg/m^2^ (IQR)	26 (23–30)	27 (24–31)	27 (24–28)	24 (22–27)	<0.001

*NWH* non-Hispanic White, *NHB* non-Hispanic Black, *NHO* non-Hispanic other, *IQR* interquartile range, *BMI* body mass index.

### Sociodemographic and lifestyle factors

Table 1 compares sociodemographic and lifestyle factors. Among Hispanics and NHBs, educational attainment was significantly lower with high school level or less in 45% and 39% of patients, respectively, compared to 25% among NHWs (*P* < 0.001). Proportionally, NHWs and NHOs had the most married patients, whereas NHBs had the most single patients (*P* < 0.001). Alcohol consumption was highest among NHWs (58% vs. 32–45% of patients, *P* < 0.001). Differences in cigarette smoking status and intensity were non-significant. Regarding BMI, overweight BMI (25–29.9) was most prevalent among Hispanics and obese BMI (≥30) among NHBs, whereas normal BMI (18.5–24.9) was most prevalent among NHOs (*P* < 0.001).

### Hematologic parameters

When stratified by race/ethnicity, no significant differences were found in the distribution of AL amyloidosis types (i.e., AL alone vs. multiple myeloma-associated AL) or the involved light-chain category (i.e., λ vs. κ) as shown in Table 2. Notably, all cases of Waldenström macroglobulinemia-associated AL amyloidosis occurred in the NHW group, with the exception of one NHB patient. While the median difference between involved (amyloidogenic) and uninvolved free light chains (dFLC) at baseline did not differ significantly by race/ethnicity, NHBs had a considerably higher proportion of patients with dFLC > 180 mg/L (39% vs. 22–33%, *P* = 0.044).

**Table 2:** Comparison of hematologic parameters by race/ethnicity.

	NHW	NHB	Hispanic	NHO	*P* value
AL amyloidosis type, *n* (%)					
AL alone	1862 (90%)	171 (89%)	76 (87%)	55 (93%)	
MM-associated AL	154 (7%)	20 (10%)	10 (12%)	3 (5%)	
Other^a^	62 (3.0%)	1 (<1%)	1 (1%)	1 (2%)	0.155
Involved light chain (*N* = 2280), *n* (%)					
κ	456 (23%)	39 (21%)	20 (24%)	13 (22%)	
λ	1492 (77%)	150 (79%)	65 (76%)	45 (78%)	0.857
dFLC^b^ (*N* = 1640)					
Median, mg/L (IQR)	89 (27–269)	116 (23–310)	76 (24–168)	90 (29–177)	0.416
≥180 mg/L, *n* (%)	455 (33%)	54 (39%)	16 (24%)	11 (22%)	0.044

*MM* multiple myeloma, *dFLC* difference between involved and uninvolved free light chains, *IQR* interquartile range.

^a^Other types included Waldenström macroglobulinemia-associated or lymphoplasmacytic lymphoma-associated AL amyloidosis.

^b^The serum free light-chain assay was unavailable before 2003.

### Organ disease characteristics

Cardiac involvement was most prevalent among NHBs (69% of patients) and renal involvement among NHWs (78% of patients), although differences in pattern of organ involvement across racial/ethnic groups did not meet statistical thresholds (Table 3). Most striking, Hispanics were found to have a significantly higher median BNP level at baseline compared to the other groups (1041 vs. 221–480 pg/mL, *P* = 0.001) with the majority having a BNP level > 700 pg/mL. This corresponded with a higher proportion of Hispanics having BU cardiac stage IIIb disease compared to NHWs (27% vs. 15% of patients, *P* = 0.027 by Fisher’s exact test), although the distribution of all cardiac stages did not differ between these two groups (*P* = 0.080). Meanwhile, NHOs had lower-stage cardiac involvement. The distribution of renal stages was similar between groups (*P* = 0.113). In this population, time intervals from first patient-reported sign or symptom (related to any involved organ) to diagnosis did not differ among groups, nor did time intervals from diagnosis to initial visit at the Amyloidosis Center (Table 3).

**Table 3:** Organ disease characteristics by race/ethnicity.

	NHW	NHB	Hispanic	NHO	*P* value
Organ involvement, *n* (%)					
Heart (*N* = 1963)	1010 (62%)	124 (69%)	56 (67%)	29 (54%)	0.122
Kidney (*N* = 2121)	1397 (78%)	134 (72%)	61 (72%)	37 (69%)	0.068
Liver (*N* = 1846)	422 (27%)	57 (32%)	21 (27%)	13 (25%)	0.508
Nervous system^a^ (*N* = 1806)	566 (38%)	51 (30%)	28 (35%)	13 (27%)	0.097
Organ biomarkers					
BNP (*N* = 1338), pg/mL (IQR)	424 (206–881)	480 (207–863)	1041 (429–1758)	221 (107–510)	0.001
BNP > 700 pg/mL, *n* (%)	212 (33%)	27 (33%)	22 (61%)	4 (17%)	0.002
BU cardiac stage^b^ (*N* = 1158), *n* (%)					
Stage I	267 (29%)	30 (26%)	18 (30%)	21 (45%)	
Stage II	400 (43%)	48 (41%)	18 (30%)	21 (45%)	
Stage III	126 (14%)	19 (16%)	8 (13%)	3 (6%)	
Stage IIIb	141 (15%)	20 (17%)	16 (27%)	2 (4%)	0.033
Pairwise comparison, *P*	Ref.	0.718	0.080	0.024	
Proteinuria (*N* = 2221), g/24 h (IQR)	1195 (105–6027)	1164 (183–4447)	486 (93–4592)	781 (103–5628)	0.059
eGFR (*N* = 2088), mL/min per 1.73 m^2^ (IQR)	68 (39–91)	73 (35–106)	64 (21–89)	90 (59–102)	<0.001
Renal stage (*N* = 1932), *n* (%)					
Stage I	787 (48%)	77 (52%)	33 (45%)	31 (57%)	
Stage II	653 (39%)	61 (42%)	34 (47%)	18 (33%)	
Stage III	218 (13%)	9 (6%)	6 (8%)	5 (9%)	0.113
Alkaline phosphatase (*N* = 2077), IU/L (IQR)	92 (71–135)	103 (71–152)	98 (76–180)	100 (74–149)	0.841
Diagnostic and referral intervals					
Time from first reported sign/symptom to diagnosis in months (*N* = 1933), median (IQR)	6.8 (2.3–13.5)	6.2 (1.7–12.7)	4.1 (1.5–9.9)	5.2 (2.0–12.8)	0.333
Time from diagnosis to initial visit at the referral center in months, median (IQR)	2.5 (1.4–6.0)	2.9 (1.4–6.4)	2.0 (1.1–4.1)	3.6 (1.6–9.7)	0.603

Laboratory measurements during initial evaluation is presented here as medians with IQRs.

*BNP* brain natriuretic peptide, *BU* Boston University, Ref. reference group, *eGFR* estimated glomerular filtration rate by CKD-EPI equation, *IQR* interquartile range.

^a^Denotes autonomic and/or peripheral nervous system.

^b^BNP and troponin-I were unavailable before the mid-2000s.

### Receipt of stem cell transplantation

**Fig. 2:**
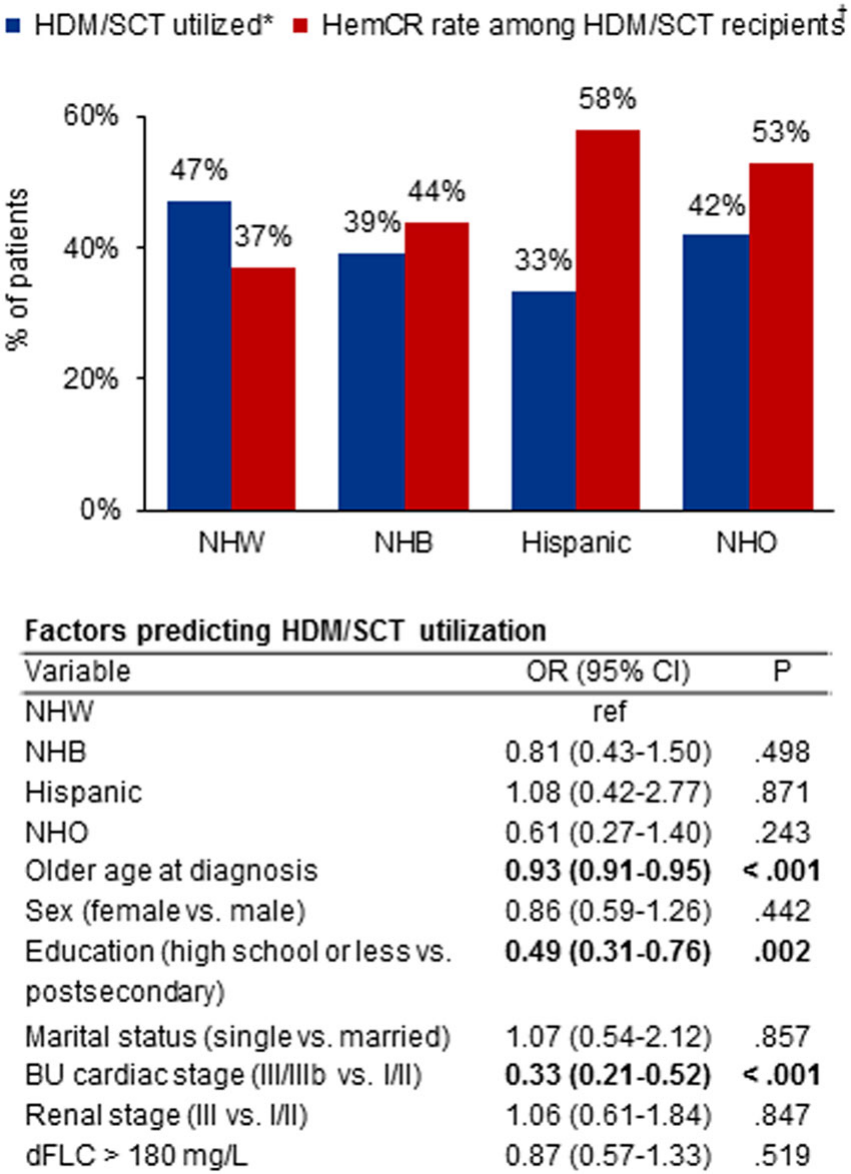
Use of HDM/SCT and post-transplantation outcomes. *Based on a subcohort of 1668 patients with available treatment data, of whom 765 received HDM/SCT. Differences in utilization were non-significant (*P* = 0.071). ^†^Differences in hematologic complete response (hemCR) rates were also non-significant (*P* = 0.562). A multivariable logistic regression analysis of HDM/SCT utilization is provided. HDM/SCT high-dose melphalan and autologous stem cell transplantation, NHW non-Hispanic White, ref reference group, NHB non-Hispanic Black, NHO non-Hispanic other, OR odds ratio, BU Boston University, dFLC difference between involved and uninvolved free light chains.

The use of high-dose melphalan and autologous stem cell transplantation (HDM/SCT) was evaluated in a subcohort of 1668 patients for whom a plasma cell-directed treatment was documented. Differences were notable, although non-significant (*P* = 0.071), between racial/ethnic groups (Fig. 2). Receipt of HDM/SCT was lowest in the Hispanic group with 19 (33%) patients transplanted, compared to 680 (47%) in the NHW, 48 (39%) in the NHB, and 18 (42%) in the NHO groups. A multivariable logistic regression, which adjusted for the effect of confounding risk factors on HDM/SCT utilization, revealed that lower educational attainment (i.e., high school level or less) and later-stage cardiac involvement (i.e., BU cardiac stage III/IIIb) significantly reduced the odds of receiving HDM/SCT, but not race/ethnicity itself. In terms of post-transplantation outcomes, rates of hematologic complete response (hemCR, assessed at 6–12 months after HDM/SCT) were non-significantly different for minority groups compared to NHWs (44–58% vs. 37%, *P* = 0.562).

### Participation in clinical trials

Between 1990 and 2020, there were 12 clinical trials of SCT and 18 of non-SCT therapies for patients with AL amyloidosis conducted at the center. Of 316 patients enrolled on SCT clinical trials (Fig. 1b), 295 (93%) were NHW, 12 (4%) were NHB, 7 (2%) were Hispanic, and 4 (1%) were NHO. Similarly, of 228 patients enrolled on non-SCT clinical trials (Fig. 1c), 199 (87%) were NHW, 18 (8%) were NHB, 7 (3%) were Hispanic, and 5 (2%) were NHO. Some patients participated in multiple trials. Collectively, representation of racial/ethnic minorities in all trials was 10%. This improved more recently, as demonstrated by the 13 trials introduced in the latest decade (2010–2020), in which 25 (22%) of 116 patients were minorities.

### Survival

**Fig. 3:**
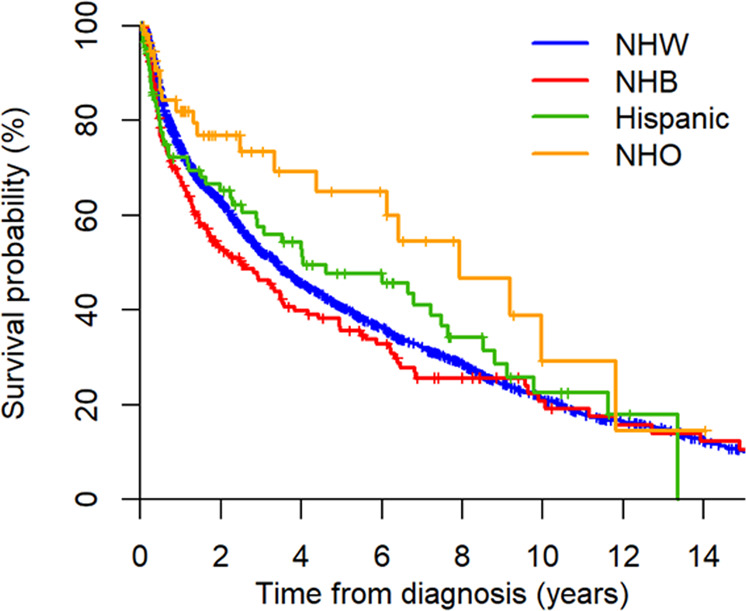
Kaplan–Meier survival curves stratified by race/ethnicity. NHW non-Hispanic White, NHB non-Hispanic Black, NHO non-Hispanic other.

At the time of data cutoff (March 2020), 1622 (67%) patients were deceased. Kaplan–Meier survival curves according to race/ethnicity are illustrated in Fig. 3. Among the groups, NHBs had the shortest median overall survival (2.5 vs. 3.4–7.9 years). Early mortality was disproportionately higher among NHBs with 23% (95% CI, 18–30%) of patients deceased within 6 months of diagnosis, compared to 15% (95% CI, 13–17%) among NHWs. Multivariate Cox proportional analysis demonstrated no significance in the overall effect of race/ethnicity on mortality; however, slight differences among specific groups were observed. The influence of various patient characteristics was accounted for in stepwise fashion (Table 4). Unadjusted, NHBs had a 15% higher HR for death than NHWs (*P* = 0.145). After adjusting for age and sex, this disparity intensified to 24% and became significant (*P* = 0.020). Adding sociodemographic and lifestyle variables to the model reduced the disparity slightly, whereas adding physiologic variables and HDM/SCT treatment diminished it entirely. The variables with the strongest effect size on mortality risk in our final analysis (model 4) were HDM/SCT receipt (HR, 0.45; 95% CI, 0.33–0.60; *P* < 0.001) and cardiac stage ≥III (HR, 1.94; 95% CI 1.48–2.54; *P* < 0.001), followed by dFLC >180 mg/mL (HR, 1.39; 95% CI, 1.06–1.82; *P* = 0.015).

**Table 4:** Multivariable Cox proportional hazard ratios for all-cause mortality with stepwise adjustments for patient characteristics.

	Median OS (years)	Unadjusted		Model 1		Model 2		Model 3		Model 4	
		HR (95% CI)	*P* value	HR (95% CI)	*P* value	HR (95% CI)	*P* value	HR (95% CI)	*P* value	HR (95% CI)	*P* value
NHW	3.4	Ref.		Ref.		Ref.		Ref.		Ref.	
NHB	2.5	1.15 (0.95–1.37)	0.145	**1.24 (1.03–1.49)**	**0.020**	1.19 (0.93–1.50)	0.161	0.92 (0.63–1.35)	0.673	0.82 (0.50–1.34)	0.427
Hispanic	4	0.92 (0.70–1.22)	0.573	0.97 (0.73–1.29)	0.846	1.05 (0.72–1.52)	0.811	0.93 (0.57–1.52)	0.082	0.60 (0.29–1.23)	0.162
NHO	**7.9**	**0.61 (0.39–0.95)**	**0.029**	0.72 (0.46–1.13)	0.151	0.61 (0.36–1.04)	0.069	0.61 (0.29–1.32)	0.210	0.70 (0.32–1.52)	0.367
Overall *P* among groups			0.059		0.050		0.133		0.631		0.377

Model 1 adjusted for demographic variables only (age, sex). Model 2 added sociodemographic and lifestyle variables (education, marital status, smoking, alcohol consumption, BMI). Model 3 added physiologic variables (BU cardiac stage, dFLC >180 mg/mL). Model 4 added stem cell transplantation treatment. The overall *P* value derived by Type III test estimated the effect of race/ethnicity in each model.

*OS* overall survival, *HR* hazard ratio, *CI* confidence interval, *ref.* reference group.

Bold values signify statistical significance.

## Discussion

While efforts have been made to understand racial/ethnic disparities in multiple myeloma and MGUS, the first steps need yet to be taken for systemic AL amyloidosis. In our analysis of 2416 patients, 334 (14%) were self-identified minorities. This percentage is considerably lower than the >36% representation of racial/ethnic minorities in the general population (based on 2010 US census data)^28^. The discrepancy was found to be most profound for Hispanics, who constitute >16% of the general population, but only represented 4% of the AL amyloidosis cohort. Although these data depict the experience of a single-referral center, the findings are compelling. Systematic underdetection may be at the root of the disparity, perhaps due in part to the misattribution of AL indicators (e.g., structural heart changes, nephrosis, neuropathy) to hypertensive disease or diabetes mellitus—both of which are more prevalent in racial/ethnic minorities.

Disparities in access to diagnostic testing and specialized treatment centers often reflect socioeconomic obstacles, which vary by race/ethnicity^29^. It was therefore important to contextualize the social determinants of health in our cohort. More adverse risk factors were observed among underrepresented minorities, including lower educational attainment and a greater proportion of unmarried individuals. Independently, these factors have important implications for health and survival. Significant differences were also noted in BMI classification—a potentially modifiable risk factor—with the Hispanic and NHB groups having disproportionately more overweight/obese individuals. Obesity and its associated low-grade inflammatory state are thought to alter plasma cell development, leading to a nearly twofold higher risk for developing MGUS and progressing to overt multiple myeloma^30–33^. Particularly among NHBs, higher body weight is associated with an excess risk of multiple myeloma-related mortality^34^. In AL amyloidosis, the role of obesity on pathogenesis of the underlying plasma cell neoplasm and/or downstream organ disease severity—which may especially impact minorities—is not yet defined.

Importantly, a number of differences in disease features were uncovered in our study. Racial/ethnic minorities were diagnosed at younger ages compared to NHWs (e.g., 4 years younger for NHBs), an observation that is paralleled in other plasma cell disorders^2,19^. This age difference was not explained by lifestyle risk exposures (i.e., alcohol use and cigarette smoking were lower or similar among minorities). Furthermore, the NHB group had disproportionately more patients with a baseline dFLC > 180 mg/L, which is thought to reflect a more aggressive disease phenotype^6^. At face value, these findings may imply unique disease physiology, and future investigations are needed to unravel the potential biologic and/or genetic underpinnings of ancestry-related disparities in AL amyloidosis. Alternatively, these findings may be explained by underdetection of NHB individuals with more indolent disease—instead necessitating heightened awareness.

Organ distribution was similar across racial/ethnic groups, yet differences in organ severity were noted. The most advanced cardiac stage (i.e., BU stage IIIb) was 1.8-fold higher among Hispanics compared to NHWs—likely a reflection of delayed disease recognition. Although the time interval from first patient-reported symptom to diagnosis was estimated not to be longer among Hispanics, it may be that some minorities underreport symptoms—due in part to language barriers—or seek medical attention later. This disparity in organ manifestation may also signal differences in the amyloidogenic light chain. While little is known about the mechanisms of organ tropism and proteotoxicity in AL amyloidosis, structural and genetically-driven features of the immunoglobulin light-chain variable region (IGLV) have been implicated. For example, germline expression of the IGLV1–44 gene has been associated with cardiac tropism, whereas the IGLV6–57 gene with renal tropism^35–37^. Variances in this gene have not before been compared across racial/ethnic populations.

When evaluating cardiac amyloidosis in persons of African ancestry, hereditary ATTR amyloidosis needs to be excluded before a diagnosis of AL amyloidosis can be established. The G > A nucleotide transition at the first base position in codon 122 of the transthyretin gene (encoding for TTR V122I) has a frequency of 3.4% among African Americans, and is a major contributor of ATTR cardiomyopathy^22^. Notably, ATTR V122I and AL amyloidosis were previously found to be equally prevalent as causes of amyloid cardiomyopathy in African Americans evaluated at our center^21^. One further challenge in establishing diagnosis is the coexistence of MGUS in nearly 50% of African Americans with ATTR V122I amyloidosis^38^. In our study, 4 (2%) of 179 patients with AL amyloidosis in the NHB group tested were found to be carriers of the V122I variant allele. Penetrance is <100% in hereditary ATTR amyloidosis; thus, not every patient who tests positive for the V122I variant allele has or will develop ATTR V122I amyloidosis. In this situation, typing of amyloid protein is essential prior to treatment. Moreover, an appropriate systemic syndrome is a requisite for AL amyloidosis, as cases of cardiac amyloid fibrils containing both light-chain and TTR proteins have recently been described^39^.

Despite an evolving therapeutic landscape for AL amyloidosis, HDM/SCT remains one of the most effective treatments available^40,41^. In our study, racial/ethnic minorities were less likely to receive treatment with HDM/SCT, with Hispanics in particular having a 30% lower likelihood compared to NHWs. Similar patterns have been reported in multiple myeloma, contributing to higher mortality risk^11–13,42^. Identifying and mitigating treatment barriers is therefore important. We found that sociodemographic (e.g., lower educational attainment) and physiologic (e.g., cardiac stage) factors were the key drivers of HDM/SCT underutilization, rather than race/ethnicity itself. This finding underscores the need for earlier disease detection in underrepresented communities—before cardiac disease becomes too advanced to qualify for HDM/SCT treatment. Moreover, tackling upstream economic barriers, expanding health literate resources, and funding care coordination programs may help bridge access to treatment with HDM/SCT. Race/ethnicity did not impact post-transplantation outcomes. A larger sample size would be needed to determine whether the 6–21% higher rates of hemCR among minorities observed in this study are significant.

Enrollment in therapeutic clinical trials can provide access to advanced treatments. Most published trials of AL amyloidosis do not report the racial/ethnic construct of the study cohort. We retrospectively evaluated the accrual of racial/ethnic minorities in 30 clinical trials conducted at our center and observed that minorities comprised only 10% of study participants. Underrepresentation of minorities in clinical trials limits understanding about the safety and efficacy of promising treatments across populations, and thus has the potential to contribute to racial/ethnic disparities^43^. Multilevel interventions are needed, including attentiveness by investigators to recruit minorities and engagement of institutions serving minority communities in trials of AL amyloidosis.

Timely diagnosis and treatment are important in order to reduce early mortality and halt organ progression in AL amyloidosis^6^. Within this referred cohort, NHBs had the highest rate of early mortality and shortest overall survival. In view of the age difference at diagnosis, mortality risk was adjusted for demographic variables and found to be significantly greater among NHBs compared to NHWs. This survival disparity was eliminated when markers of disease severity (i.e., cardiac stage and dFLC) were taken into account, indicating that race itself did not impact survival. Interestingly, Hispanics were not found to have inferior survival despite more patients having stage IIIb cardiac involvement—perhaps due to insufficient sample size to detect a difference or more indolent disease phenotype in this population.

There are several limitations of our study. Race and ethnicity are sociocultural—rather than biological—constructs. Self-reported racial/ethnic category can therefore be a biased measure when evaluating differences in disease characteristics. Another inherent limitation is referral bias. Underprivileged individuals face greater difficulties in accessing tertiary centers, and thus may remain uncaptured in our study. Large population databases of AL amyloidosis apart from referral centers, however, are nonexistent. Furthermore, the unavailability of a direct measure of socioeconomic status in our database—which is an important influence in studies of race/ethnicity—required us to depend on surrogates, such as educational level. Despite these limitations, this study sheds light on the clinical presentation and outcomes of minority patients with AL amyloidosis who are underrepresented in the medical literature to date.

## Conclusions

Racial/ethnic minorities comprised a small portion of patients with systemic AL amyloidosis seen at a referral center, drawing attention to systematic barriers in health care access. The minorities captured in this study already overcame many of the obstacles that underlie disparities. Nonetheless, earlier and more severe diagnosis was detected among NHBs and Hispanics. Cardiac staging and educational level accounted for underutilization of HDM/SCT, rather than race/ethnicity itself. After controlling for disease severity and HDM/SCT treatment, survival did not differ by race/ethnicity. Greater awareness is needed, along with innovative approaches to make services and technologies for earlier identification of AL amyloidosis available in underrepresented communities.

## Supplementary information

Supplementary information

## Supplementary information

**Supplementary Information** accompanies this paper at (10.1038/s41408-020-00385-0) .
